# SlBEL11 affects tomato carotenoid accumulation by regulating *SlLCY-b2*

**DOI:** 10.3389/fnut.2022.1062006

**Published:** 2022-12-22

**Authors:** Yan He, Yu Wang, Mengzhuo Zhang, Guangsen Liu, Cong Tian, Xiangbin Xu, Yonggui Pan, Xuequn Shi, Zhengke Zhang, Lanhuan Meng

**Affiliations:** ^1^School of Food Science and Engineering, Hainan University, Haikou, China; ^2^Key Laboratory of Food Nutrition and Functional Food of Hainan Province, Haikou, China

**Keywords:** BEL1-LIKE HOMEODOMAIN 11, carotenoids, transcription factor, tomato fruit, virus induced gene silencing

## Abstract

Extensive data have demonstrated that carotenoid accumulation in tomato fruit is influenced by environmental cues and hormonal signals. However, there is insufficient information on the mechanism of its transcriptional regulation, as many molecular roles of carotenoid biosynthetic pathways remain unknown. In this work, we found that the silence of the BEL1-like family transcription factor (TF) BEL1-LIKE HOMEODOMAIN 11 (SlBEL11) enhanced carotenoid accumulation in virus induced gene silencing (VIGS) analysis. In its RNA interference (RNAi) transgenic lines, a significant increase in the transcription level for the lycopene beta cyclase 2 (*SlLCY-b2*) gene was detected, which encoded a key enzyme located at the downstream branch of the carotenoid biosynthetic pathway. In Electrophoretic mobility shift assay (EMSA), SlBEL11 protein was confirmed to bind to the promoter of *SlLCY-b2* gene. In addition, the dual-luciferase reporter assay showed its intrinsic transcriptional repression activity. Collectively, our findings added a new member to the carotenoid transcriptional regulatory network and expanded the functions of the SlBEL11 transcription factor.

## Introduction

In tomato (*Solanum lycopersicum*), degradation of chlorophyll and accumulation of carotenoids result in the red color of ripe tomato fruits. Carotenoids are a group of important natural pigments, present not only in plants, but also in algae, fungi, and bacteria (1–3). In plants, carotenoids serve numerous crucial functions. Notably, carotenoids are essential for photosynthesis of photosynthetic organisms, and also play a vital role in protecting photosynthetic organisms under excessive light conditions (4). Carotenoids also are an important source of phytohormones including abscisic acid (ABA), which is involved in many aspects of plant developmental processes including inducing seed dormancy and responding to abiotic stresses (5). Studies have also provided evidence that carotenoid derivatives mediate plant development and act as signaling molecules in response to environmental factors including strigolactones, β-cyclocitral, and dihydroactinidiolide, a mobile bypass signal and karrikins with structural similarity to strigolactones (6–8). Carotenoids also exert biologically important functions in humans. Carotenoids are the main dietary source of vitamin A and also provide antioxidants to reduce the risk of cancer and alleviate chronic diseases (9, 10). In particular, β-carotene, lycopene, lutein and zeaxanthin have been widely identified as biological antioxidants and immune system modulators. The potential protective effect of high β-carotene intake in the development of cardiovascular diseases is also supported by earlier epidemiological studies (11). There have also been a large body of researches about carotenoids and their effects on cardiovascular diseases and infectious diseases such as HIV infections (12). As the main constituents of macular pigment, lutein and zeaxanthin are important for eye health. Their accumulation in the retina reduces the risk of macular degeneration, which is responsible for the main cause of blindness in the elderly (13).

Researches into carotenoids and their functions in human health have been paralleled by major advances in cloning core genes involved in carotenoid production. Taken together, it is these cognitive advancements that have ushered in a new era of elevated carotenoid levels in food. At present, highly conserved carotenoid biosynthetic pathway has been resolved in many plants (14). The major source of biosynthesis carotenoid molecules is methylerythritol-4-phosphate (MEP) pathway in plants (15). Geranylgeranyl diphosphate (GGPP) is ubiquitous and it is the isoprenoid precursor to generate carotenoids. GGPP is converted to phytoene, which is catalyzed by the phytoene synthase (*PSY*), a rate-limiting enzyme. This step has been subjected to genetic regulation (1). In tomato, both *PSY1* and *PSY2* are present in the fruit, but *PSY1* functions in the formation of carotenoids during fruit ripening, while *PSY2* primarily works in the formation of carotenoids in chloroplast-containing tissues (16). Then, the phytoene synthase catalyzed product, phytoene is catalyzed sequentially into ζ-carotene and prolycopene by the enzymes phytoene desaturase (*PDS*) and ζ-carotene desaturase (*ZDS*), respectively (14). Isomerization of prolycopene to red-colored lycopene is carried out by carotene isomerase (*CRTISO*). Lycopene is further metabolized to produce β-carotene, zeaxanthin, violaxanthin, and neoxanthin, which is catalyzed by two classes of lycopene beta cyclases (*LCY-bs*, *CYC-b*). Specifically, *LCYbs* are thought to play a critical key role at the branching point of the carotenoid biosynthetic pathway, including the conversion of red lycopene to the downstream bright yellow or orange lutein (17). The existence of two types of *LCY-bs* genes have been confirmed in many plants. Notably, *LCY-b1* is mainly expressed in leaf tissue, while *LCY-b2* is primarily expressed in fruit tissue (18). Studies have demonstrated that altered expression levels of *LCYbs* cause abnormal lycopene accumulation in fruits or flowers (19). It has been reported that lycopene accumulation in red grapefruit *(Citrus paradisi*) results from downregulation of the citrus chromosomal-specific *LCY-b2* (17). In citrus calli and tomato fruits, the overexpression of CsERF061 increases carotenoid accumulation and promotes fruits growth through directly binding to the promoter of *LCY-b2* (20). Although the function of the promoter of *LCY-b1* has been characterized in tomato, little information is available on the *LCY-b2* promoter.

Numerous studies have proved that carotenoid biosynthesis is regulated by a variety of transcription factor families, including the R2R3-MYB, MADS-box, NAC, bHLH, SBP-box, AP2/ERF, HD-ZIP, NF-Y, and WRKY (21–24). The spontaneous ripening-related mutants in tomato, such as ripening-inhibitor *(Rin)*, non-ripening (*Nor*), and colorless non-ripening *(Cnr)* promote our current understandings of the mechanisms underlying fruit ripening (25). Mutations at these loci can completely block normal ripening of tomato fruit. NOR-LIKE1 transcription factor is involved in lycopene accumulation by directly binding to *SlSGR1* and *SlGgpps2* genes and activating their expression (25). Overexpression of *SlAPRR2* and *SlGLK1* genes in tomato increased the number of plastids in fruit and carotenoid accumulation in fruit at ripening stage was higher than that of wild type (22). Silencing of *SlNAC1* in tomato resulted in up-regulation of the expression of *SIPSY1* and accumulation of total carotenoids. Besides, carotenoid accumulation was positively regulated by binding *SlPSY1*, *SlZ-ISO*, *SlCRTISO* promoters (23, 26, 27). However, the evaluation of RIN binding to promoters of target genes in ChIP experiments yielded inconsistent results (28) and the expression of *SlPSY1* in FUL1-silenced and FUL2-silenced tomato fruits was not altered (29). Therefore, the roles of some transcription factors in regulating carotenoid biosynthesis still need to be further explored. A lot of regulatory factors of upstream products have been identified in the carotenoid metabolic pathway, while the research data of transcriptional regulation of downstream substances is insufficient.

In our experiment, the TF *SlBEL11* gene had high expression level during ripening in tomato fruit. *SlBEL11* silencing led to a pronounced immature phenotype, with fruit not reddening normally. The experimental results revealed that SlBEL11 was involved in the carotenoid accumulation of tomato fruit at the red stage by directly binding to the promoter of the *LCY-b2* gene. Our results provided some evidence about the transcriptional regulation of tomato fruit carotenoid metabolism.

## Materials and methods

### Plant material

Tomato plants (*Solanum lycopersicum* cv. Micro Tom) and *BEL11 RNAi* transgenic plants from the previous study (30) were grown in a greenhouse at 25°C with 16 h light and 8 h dark alternating. Flowers were tagged at anthesis. Clean and healthy fruit samples were immediately used for experiments or stored at –80°C after quick freezing in liquid nitrogen until they were used for measurement.

### Virus induced gene silencing (VIGS)

pTRV1 and pTRV2 vectors were subjected to this experiment described by Liu et al. (31). A 420-bp specific fragment of the *SlBEL11* gene corresponding to bases 778–1197 of the *SlBEL11* gene, a 540-bp fragment of the *SlPDS* gene located in bases 764–1303 of the *PDS* gene, a 340-bp fragment of the *SlLCY-b1* gene located in bases 537–876 of the *SlLCY-b1* gene, and a 339-bp fragment of the *SlLCY-b2* gene located in bases 571–909 of the *SlLCY-b2* gene were PCR amplified from tomato cDNA using Kod-plus polymerase (TOYOBO, Japan) and primers shown in Supplementary Table 1. The PCR amplified products then were ligated into the pTRV2 plasmids which were digested by *Sac*I and *Xho*I. Then, pTRV1 vector plasmid and the recombinant plasmids identified by sequencing were transferred into *Agrobacterium tumefaciens* strain GV3101. The preparation of agrobacterium infection solution and the method of infecting tomato sprouts were described by Meng et al. (30). Tomato sprouts infiltrated with empty pTRV2 and pTRV2-*PDS* were set as negative and positive controls (31).

### Real-time quantificational PCR (qRT-PCR) for gene expression analysis

The extraction of total RNA was completed according to the manufacturers’ method described by the RNAprep Pure Plant Kit (TIANGEN Biotech, Hainan, China). In this experiment, containers and consumables were treated with RNase inactivation to ensure the integrity of the extracted RNA. The next steps were required to be completed on ice to confirm that the samples were not degraded. The RNA was subjected to cDNA synthesis with Fastking One Step RT-PCR Kit (TIANGEN Biotech, Hainan, China) following the instructions of the reagent manufacturer. qRT-PCR was conducted to examine the mRNA levels of the genes. In total, 1 μL of cDNA, together with 5 μL of SYBR green mix (TIANGEN Biotech, Hainan, China), 0.3 μL each forward and reverse primer, were added into the PCR mixtures (with the total volume of 10 μL). The PCR reactions were performed in A CFX Connect Detection System (Bio-Rad, CA, USA). *Actin* was selected as the reference gene. The method of 2^–ΔΔCt^ was adopted for calculation of results. The primer sequences were provided in Supplementary Table 1.

### Analysis of carotenoid content by HPLC

Fresh samples were pulverized with liquid nitrogen. In total, 50 (±1) mg of sample powder was extracted for 24 h with 1 mL of extraction solution (methanol:water:formic acid = 75:23:2) and vortexed for 15 min. After centrifugation, add 0.5 ml extraction solution to the residue, repeat shaking and centrifugation, and combine the supernatant. Concentrate and dry in the concentrator at 4°C, reconstitute with 200 μL 80% methanol. Centrifuge again, store the supernatant in the bottle for LC-MS/MS analysis. The mobile phase consisted of 0.04% acetic acid in water (solvent A) and 0.04% acetic acid in acetonitrile (solvent B). The flow rate was 0.4 ml/min and the column temperature was 40°C. The parameters of the gradient separation were as follows: increase 5% B–95% B in 10 min, hold for 1 min and change back to a 5% B concentration in 6 s, hold for 2.9 min.

### Electrophoretic mobility shift assay (EMSA)

The coding sequence of the SlBEL11 HD-box was PCR amplified. Then, the coding sequence was cloned into pGEX-4T-1 which was fused with a glutathione S-transferase (GST) tag. The recombinant construct identified by sequencing was transformed into *Escherichia coli* strain Rosetta (DE3). Protein expression and purification were completed referring to the method reported by Xiao et al. (32).

The probes containing **TTGACTTGAC**atagt**GTCA** motif sequence from the promoter of *SlLCY*-*b2* were biotinylated using a DNA 3′ End Biotinylation Kit (Thermo Fisher Scientific, Waltham, USA). The same sequence without labeled DNA fragment was a competitor. The **TTGACTTGAC**atagt**GTCA** DNA fragments were modified into **TTTTTTTTTT**atagt**TTTT** and used as a mutant probe in the assay. EMSA was performed according to the manufacturer’s protocol provided by A LightShift Chemiluminescent EMSA kit (Thermo Scientific).

### Dual-luciferase reporter assay

In this assay, the *SlLCY-b2* promoter was cloned into the pGreenII 0800-LUC dual reporter gene vector (33). The *SlBEL11* gene was cloned into the pEAQ vector (34) as effector. The constructed *SlLCY-b2-pro-*LUC effector and pEAQ-HT-SlBEL11 reporter plasmids were co-transformation of tobacco with *Agrobacterium tumefaciens* strain GV3101. The method was subject to Yin et al. (35). The *Agrobacterium* cells that has been transformed into the corresponding expression vector were inserted into the LB liquid medium containing kanamycin and gentamicin for cultivation (28°C, 240 rpm, 12–24 h). The *Agrobacterium* cells were collected and suspended in osmotic solution (10 mM MES, 10 mM MgCl_2_, and 150 μM acetosyringone, pH 5.6), and adjusted to an appropriate concentration. The bacteria containing effector and reporter were mixed in a ratio of 9:1, and then the mixture was injected into tobacco leaves and cultured for 2–3 days. The ratio LUC/REN of two luciferase enzymes (LUC and REN) in leaves was determined by the dual luciferase reporting and analysis system (Promega). Each assay should contain at least 6 measurements.

### Statistical treatment of data

The significance between the data was analyzed by *t*-test. The bar and line charts were plotted using GraphPad Prism 9 software.

## Results

### Silencing of SlBEL11 affects carotenoid accumulation in tomato fruit

**FIGURE 1:**
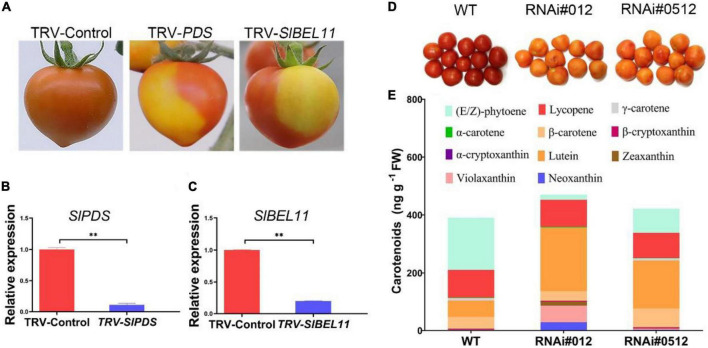
Silencing of the *SlBEL11* gene affects carotenoid accumulation in tomato fruits. **(A)** Ripe fruit of plants infected with empty vector virus (TRV-Control), specific fragment of *SlPDS* gene (TRV-SlPDS), and specific fragment of *SlBEL11* gene (TRV-*SlBEL11*). **(B)** Analysis of qRT-PCR for *SlPDS* gene in tomato fruits in TRV-Control (red sections) and TRV-*SlPDS* group (yellow sections). **(C)** qRT-PCR analysis for *SlBEL11* gene in tomato fruits in TRV-Control (red sections) and TRV-*SlBEL11* group (yellow sections). The internal reference was *actin* gene. The results were presented mean ± standard deviation (SD) of three independent replicates. **Indicated a *P*-value < 0.01 (Student’s *t*-test). **(D)** Tomato fruit phenotype of *SlBEL11 RNAi* and wild type (WT) at the red stage. The stage of tomato was defined by external and internal color of the tomato fruit. For the mature green stage, fruits will ripen to an acceptable level of horticultural quality. The entire surface of the fruits was either green or white, with no visible red. At the red stage, there was more than 90% of the surface showing red color. **(E)** Carotenoids content in *SlBEL11 RNAi* and WT tomato fruits determined at 5 days after breaker (Br + 5) stage.

The SlBEL11 belongs to the BEL1-like Homeodomain (BEL1-like) TFs family with high expression abundance in the red stage and involved in the regulation of chlorophyll synthesis and fruit pigmentation in tomato fruit (30). Compared with the control fruit, in addition to the observation that the *SlBEL11 RNAi* tomato fruits accumulate more chlorophyll at mature green stage, we also found that it still remained yellow at the red stage (Figure 1D). To further explore its possible role in tomato fruit, virus-induced gene silencing (VIGS) analysis was executed. In this experiment, a clearly demarcated red-yellow region was detected in fruits infected with the virus containing the TRV-*SlPDS* vector compared to tobacco rattle virus (TRV)-infected control and even red fruits. Among them, the tomato fruit injected with TRV-*SlPDS* was used as a positive control (Figure 1A). Similar to TRV-*SlPDS* tomato, tomato fruits injected with TRV-*SlBEL11*-containing vector virus also displayed mottled yellow and red areas, which were also separated by distinct borders. Gene expression analysis using quantitative real-time PCR (qRT-PCR) showed a reduction of approximately 80 and 90%, respectively, in *SlPDS* and *SlBEL11* transcripts in the yellow areas of the TRV-*SlPDS* and TRV-*SlBEL11* fruits, compared with the red TRV-Control fruits (Figures 1B, C). This mean that SlBEL11 may be involved in the regulation of carotenoid synthesis in tomato fruit.

In order to verify the hypothesis, we first carried out experiments to determine whether there was a difference in carotenoid content in the *SlBEL11 RNAi* and WT tomatoes. In this study, we found that the total carotenoid content in *SlBEL11 RNAi* tomatoes were much higher than that of WT at Br + 5 stage (Table 1). Compared to WT fruits, RNAi#012 and RNAi#0512 fruits showed a 90 and 54% reduction in phytoene, which was derived from upstream of the carotenoid synthesis pathway, respectively (Table 1). Whereas, the accumulation of substances downstream of carotenoid metabolism pathway was more than that of WT (Figure 1E). It is worth mentioning that large amounts of lutein accumulated in *SlBEL11* silencing fruits and the content of lutein estimated in RNAi#012 (218.65 ng⋅g^–1^) and RNAi#0512 fruits (166.67 ng⋅g^–1^), was four times and three times that of WT fruits (56.67 ng⋅g^–1^), respectively. Though the *SlBEL11 RNAi* tomatoes showed comparable lycopene content to that of WT plants, the silencing of *SlBEL11* gene resulted in a large increase of carotenoids including zeaxanthin, violaxanthin, and neoxanthin in *SlBEL11 RNAi* tomatoes. Remarkably, the most striking is that the accumulation of violaxanthin and neoxanthin in the RNAi#012 tomato fruits was detected to be approximately 42 and 35 times that of WT, respectively. Overall, the loss of *SlBEL11* function tremendously affected carotenoid metabolism, which led to abnormalities in the color of the tomato fruit.

**TABLE 1:** Carotenoids content of *SlBEL11 RNAi* and WT tomato fruit.

Compounds	Carotenoids contents (ng⋅g^–1^ FW)		
	WT	*RNAi*#012	*RNAi*#0512
(E/Z)-phytoene	179.44 + 0.25a	18.19 + 0.50c	83.23 + 0.96b
Lycopene	96.56 + 11.40a	93.03 + 9.87a	86.49 + 8.80a
γ-carotene	8.39 + 0.43a	0.72 + 0.06b	7.63 + 0.54a
α-carotene	2.68 + 0.10a	1.48 + 0.20b	1.16 + 0.07c
β-carotene	41.22 + 0.74b	33.84 + 0.3c	64.64 + 2.30a
β-cryptoxanthin	2.56 + 0.11b	3.33 + 0.10a	3.33 + 0.14a
α-cryptoxanthin	0.55 + 0.01a	0.17 + 0.02c	0.46 + 0.01b
Lutein	56.67 + 2.26c	218.65 + 7.54a	166.67 + 6.52b
Zeaxanthin	0.96 + 0.03b	13.27 + 0.39a	1.27 + 0.02b
Violaxanthin	1.37 + 0.04c	57.61 + 1.99a	3.72 + 0.16b
Neoxanthin	0.82 + 0.09b	28.74 + 3.18a	2.83 + 0.14b
Total carotenoids	390.04 + 15.45c	470.23 + 24.17a	421.43 + 19.67b

Carotenoids were tested in *SlBEL11 RNAi* and WT fruits at Br + 5 days. Three representative fruits at least from each plant were used for every measurement. The results were presented mean ± SD. FW, fresh weight. The significance was represented by different letters (*P* < 0.05).

### Silencing of SlBEL11 alters the expression of enzyme genes in the carotenoid biosynthetic pathway

**FIGURE 2:**
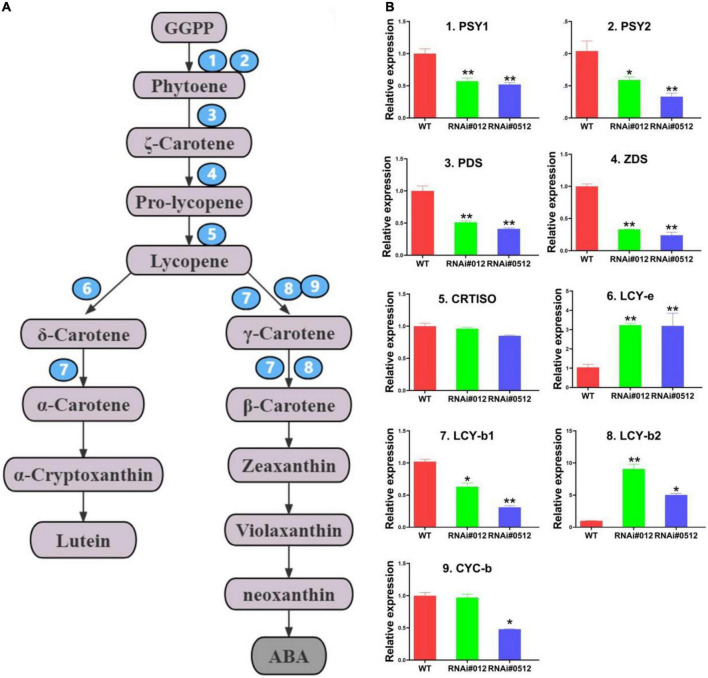
Relative transcript levels of genes related to carotenoids metabolic pathway from WT, RNAi#012, and RNAi#0512 tomato fruits in Br + 3 stage. **(A)** Schematic representation of the carotenoid metabolic pathway. *Carotene isomerase*; *LCY-e*. Abbreviations for other metabolites and enzymes have been described in the section “Introduction.” **(B)** Relative expression of core enzyme genes in carotenoids metabolic pathway in WT, RNAi#012, and RNAi#0512 tomatoes at Br + 3. Data contained three biological replicates, *representing *P* < 0.05 and **representing *P* < 0.01 (*t*-test).

To explain how SlBEL11 regulated carotenoid synthesis at the genetic level, we examined the transcript levels of main enzyme genes from carotenoid biosynthesis pathway (*PSY1*, *PSY2*, *PDS*, *ZDS*, *CRTISO*, *LCY-b1/b2*, *CYC-b*, and *LCY-e)* by qRT-PCR (Figure 2). In the *SlBEL11 RNAi* tomato lines, the expression levels of *PSY1*, *PSY2, PDS*, and *ZDS* were much lower than those in the WT line (Figure 2B). These enzyme genes mainly came from the upstream of carotenoid biosynthesis pathway. On the contrary, the expression levels of *LCY-b2* and *LCY-e* genes, which were derived from the downstream of the carotenoid metabolic pathway, were detected to be strikingly increased in the *SlBEL11 RNAi* tomato lines. Remarkably, the expression level of *LCY-b2* transcript was elevated about nine and fivefold in RNAi#012 and RNAi#0512 tomato fruits respectively, compared to WT fruits. The obvious up-regulation of the *LCY-b2* gene was consistent with the marked increase in the content of its catalytic products especially zeaxanthin, violaxanthin and neoxanthin. Similarly, the transcriptional level of *LCY-e* increased by threefold in both RNAi#012 and RNAi#0512 transgenic lines, which may be the reason for the massive accumulation of lutein downstream of the carotenoid metabolism pathway. Hence, silencing of *SlBEL11* in tomato enhanced carotenoid content downstream, which may be caused by its transcriptional regulation of carotenoid pathway genes.

### SlBEL11 directly binds the promoter of *LCY-b2*

**FIGURE 3:**
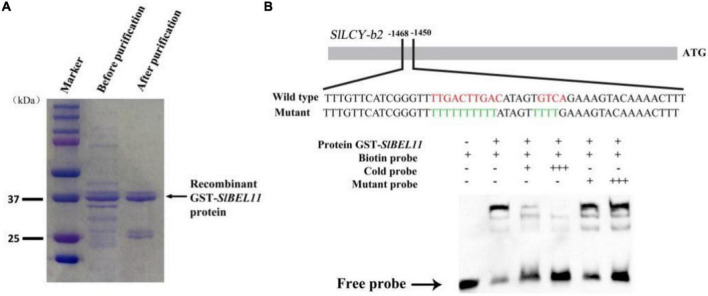
SlBEL11 bound to the promoter of *SlLCY*-*b2* gene *in vitro*. **(A)** Affinity purification of recombinant GST-*SlBEL11* protein by SDS-PAGE gel. **(B)** SlBEL11 bound to the promoter of *SlLCY*-*b2* gene containing **TTGACTTGAC**atagt**GTCA** element in EMSA. + and – indicate the presence and absence of the indicated probe or protein, respectively.

To further explore the potential regulatory mechanisms of increased content of specific carotenoids and upregulation of specific genes from carotenoid biosynthetic pathway in *SlBEL11 RNAi* lines, we performed electrophoretic mobility-shift assay (EMSA). We found one possible motif **TTGACTTGAC**atagt**GTCA** in the *LCY-b2* promoter (Figure 3B). In EMSA, the capacity of the SlBEL11 protein of regulating the *LCY-b2* promoter was analyzed. As shown in Figure 3, recombinant SlBEL11 protein physically bound to the *LCY-b2* promoter fragment and caused changes in mobility. After adding more untagged competitor protein with the same sequence, the shifted band disappeared. In contrast, GST-*SlBEL11* still showed bands with labeled probes when excess mutant probe was added (Figure 3B). Overall, in this experiment, SlBEL11 can directly bind to the **TTGACTTGAC**atagt**GTCA** motif in the *LCY-b2* promoter.

### SlBEL11 inhibits promoter activity of *LCY-b2*

**FIGURE 4:**
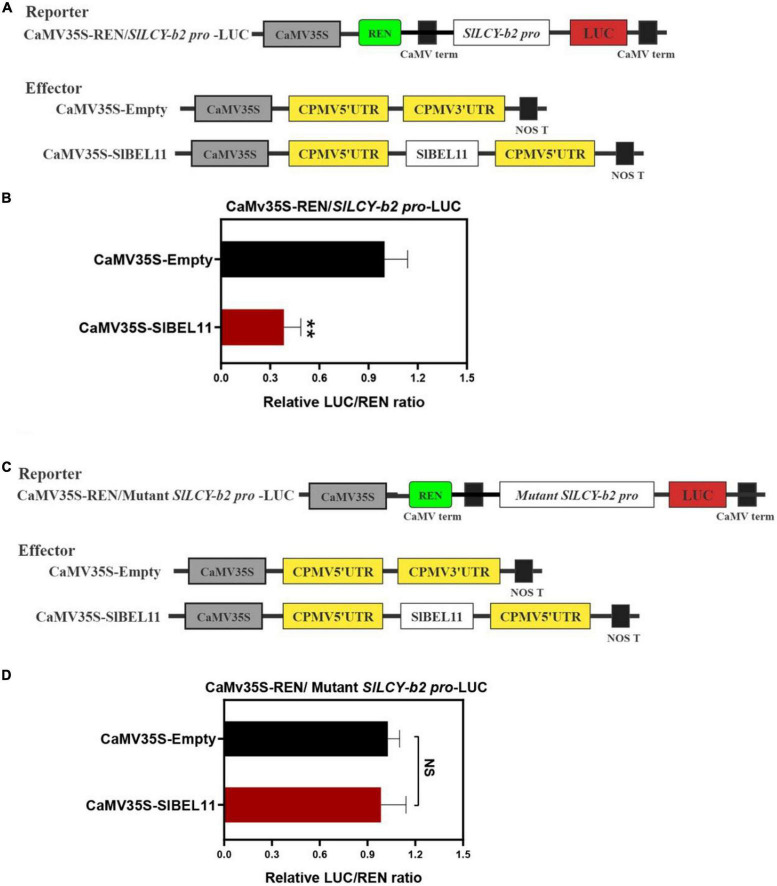
The dual luciferase reporter assay on SlBEL11 transcriptional repression of *SlLCY-b2*. **(A,C)**, Schematic representation of reporters and effectors vector plasmids. **(B)** SlBEL11 suppressed the promoter activity of *SlLCY-b2.* **(D)** SlBEL11 can not suppress the activity of mutant *SlLCY-b2* promoter. Values showed the means of six biological replicates, error bars represented SE. **Indicated that the difference between the values was extremely significant (*P* < 0.01) by Student’s *t*-test. NS, no significant difference.

Through EMSA experiment, it has been determined that SlBEL11 can bind *LCY-b2* promoter. But whether it has the activity of inhibiting or activating the *LCY-b2* gene promoter needs to be further determined. To this end, the dual luciferase reporter assays were performed to examine whether it functioned to repress the *LCY-b2* gene promoter. The dual luciferase reporter plasmids contained the *SlLCY-b2* promoter were fused to LUC, which contained the **TTGACTTGAC**atagt**GTCA** element. The CaMV35S promoter-driven REN served as an internal control, while the pEAQ-HT-SlBEL11 plasmid was used as an effector (Figure 4A). Compared with the control, expression of SlBEL11 significantly repressed the promoter activity of *SlLCY-b2* (Figure 4B). Intriguingly, when the **TTGACTTGAC**atagt**GTCA** element in promoter of *SlLCY-b2* was mutated into **TTTTTTTTTT**atagt**TTTT** sequence (Figure 4C), there was no significant change in LUC/REN ratio (Figure 4D). From this we can conclude that the **TTGACTTGAC**atagt**GTCA** element underlay the transcriptional repressive activity of SlBEL11 on the *SlLCY-b2* gene promoter.

### VIGS analysis of *SlLCY-b2*

**FIGURE 5:**
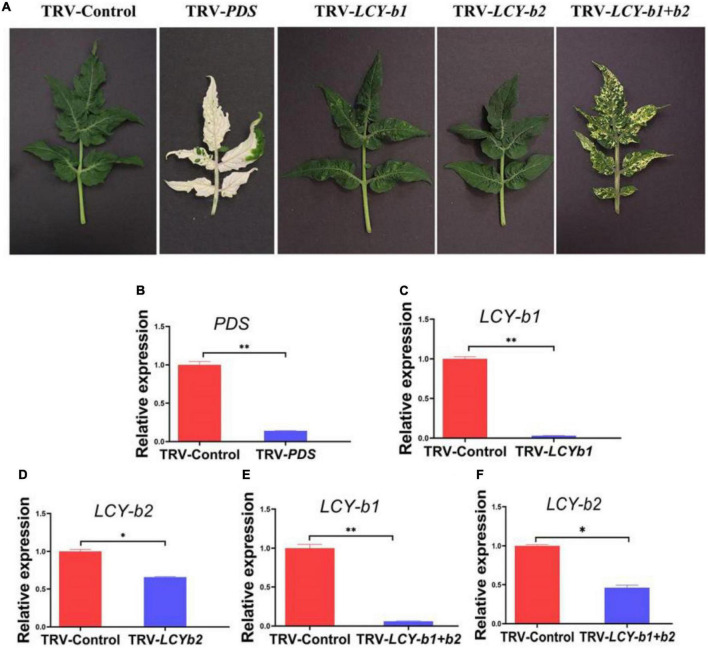
VIGS analysis of *LCY-bs.* **(A)** The excised leaves of 6-week-old tomato plants infected separately with vectors containing no insert (TRV-Control), specific sequences (TRV-*PDS*; TRV-*SlLCY-b1;* TRV-*SlLCY-b2:* TRV-*SlLCY-b1* + *b2)*. **(B)** Analysis of relative gene expression for *PDS* gene of tomato leaves in TRV-Control and TRV-*PDS* groups. **(C)** Analysis of relative gene expression for *LCY-b1* gene in tomato leaves in TRV-Control and TRV-*LCY-b1* groups. **(D)** Analysis of relative gene expression for *LCY-b2* gene in tomato leaves in TRV-Control and TRV-*LCY-b2* groups. **(E)** Analysis of relative gene expression for *LCY-b1* gene in tomato leaves in TRV-Control and TRV- *LCY-b1* + *b2* groups. **(F)** Analysis of relative gene expression for *LCY-b2* gene in tomato leaves in TRV-Control and TRV-*LCY-b1* + *b2* groups. The internal reference was *actin* gene. The values were expressed as the mean ± SD of three independent repeats. *Indicated a *P*-value < 0.05; ^**^Indicated a *P*-value < 0.01 (*t-*test).

Comparison of the amino acid sequence of *SlLCY-b2* with known lycopene beta cyclase homologous protein sequences revealed a high degree of homology to other *LCY-bs* (Supplementary Figure 1). Particularly, *SlLCY-b2* showed 87% identity with the amino acid sequence of *SlLCY-b1*, which predicted functional similarity between the two genes. Studies have reported that up-regulation of the *SlLCY-b1* gene caused fruit orange pigmentation, which was accompanied by increased β-carotene content. Further VIGS experiment was conducted to investigate the possible function of *SlLCY-b2.* In this experiment, silencing of the positive control *PDS* gene in Micro-Tom tomato plants results in aberrant carotenoid biosynthesis, conferring a leaf photo-bleaching phenotype (Figure 5A), which ensured that TRV clones caused gene silencing. Tomato infected with TRV-*SlLCY-b1* developed a yellow spotted phenotype in leaves about 6-week post-Agro-infiltration (Figure 5A). When *SlLCY-b1* and *SlLCY-b2* genes were silenced at the same time, tomato leaves developed a more pronounced yellow phenotype, suggesting that carotenoid synthesis was affected seriously (Figure 5A). However, no apparent phenotype was detected in tomato leaves infiltrated with TRV-*SlLCY-b2*. Then, RT-qPCR was performed to analyze the effect of VIGS on *SlPDS*, *SlLCY-b1*, *SlLCY-b2*. In tomato plants infiltrated with TRV-*PDS*, the transcript level of the *PDS* gene were detected to be reduced by 96% compared with the TRV infected controls, which was consistent with the photo-bleaching phenotype of *PDS*-silenced tomato plants (Figures 5A, B). Contrasted with TRV-Control plants, the gene expression of *LCY-b1* in the leaves of TRV-*LCYb1* tomato plants was down-regulated by about 99%, which was comparable to the expression level of *LCY-b1* in TRV-*LCYb1* + *b2* plants (Figures 5C, E). However, in TRV-*LCYb2* tomato plants, the expression level of the *LCYb2* gene changed slightly, with a silencing level of 34%, which may result in invisible phenotype in its leaves (Figure 5D). In TRV-*LCYb1* + *b2* tomato plants, *LCY-b1* and *LCY-b2* genes were silenced to varying degrees, and the silencing efficiencies were 99% and 54%, respectively, relative to the control plants (Figures 5E, F). These results may suggest that down-regulation of the *LCY-b2* gene, resulting in inhibition of carotenoid biosynthesis, was partly responsible for the yellowing phenotype of TRV-*LCYb1* + *b2* tomato plants.

## Discussion

The BEL1-like family (BELL) of transcription factors is ubiquitous in plant and typically plays important roles in cell wall formation, flower and leaf development (36). Previous studies reported that the silence of SlBEL11 resulted in the dark green phenotype of immature tomato fruit, which explained its transcriptional regulation role in chloroplast development and chlorophyll synthesis (30). However, there is no evidence that SlBEL11 regulates carotenoid accumulation of tomato fruits. A total of 13 BEL1-like transcription factors expressed in various organs and developmental stages in tomato, among which SlBEL11 has a higher expression level during ripening stage, which indicates that it has a certain function at the ripening process. In our experiment, it was observed that *SlBEL11* gene distinctly affected fruit coloration when the ripening candidate transcription factor SlBEL11 was silenced by VIGS approach. Therefore, it was speculated that SlBEL11 might be involved in the regulation of genes related to pigment formation, or the expression of ripening genes, or the hormone signal transduction process. Later transgenic tomatoes confirmed SlBEL11 as a transcriptional regulator of tomato fruit carotenoid accumulation.

In our work, we found that *SlBEL11 RNAi* tomato fruits were yellow at the red stage, while WT tomato fruits were all red, suggesting that silencing of *SlBEL11* gene affected fruit pigment accumulation (Figure 1D). Further experiments revealed that the total amount of major carotenoids was elevated in *SlBEL11 RNAi* tomato fruits (Table 1). There were many kinds of carotenoids, among which red lycopene and yellow β-carotene are dominant in tomato fruit (37, 38). Our results demonstrate that silencing of *SlBEL11* gene resulted in a decrease in upstream carotenoids and an increase in downstream carotenoids (Figure 1E). It has been reported that the accumulation of fruit pigments was determined by the expression of genes related to carotenoid synthesis (39). Compared to WT, phytoene was detected considerably dropped in *BEL11 RNAi* tomato fruits (Table 1), which may be due to the remarkable decrease in the expression of *PSY1* and *PSY2* in the carotenoid synthesis pathway. The increased lutein, zeaxanthin, violaxanthin and neoxanthin in *SlBEL11 RNAi* fruits may provide explanation for the yellow phenotype of *SlBEL11 RNAi* fruits. Lutein, the product of lycopene catalyzed by *LCY-e* enzyme, increased threefold in *SlBEL11 RNAi* fruits relative to WT (Figure 2). The increase of zeaxanthin, violaxanthin and neoxanthin may be caused by the elevation of *LCY-b2*, which were nine and fivefold higher in RNAi#012 and RNAi#0512 than that in WT, respectively. Previous studies have demonstrated the down-regulation of *SlLCY-b1* gene was the mechanism responsible for the massive accumulation of lycopene during fruit ripening, which turned the tomato’s red fruit to orange (40, 41). To date, however, the roles of *SlLCYb2* in carotenoid biosynthesis are still unclear. *SlLCY-b2* showed 87% identity with the amino acid sequence of *SlLCY-b1* (Supplementary Figure 1), which predicted similar functions between *SlLCY-b1* and *SlLCY-b2*. In VIGS experiment, the co-silencing of *SlLCY-b1* and *SlLCY-b2* genes severely affected the production of carotenoids in tomato leaves, resulting in yellow phenotype in leaves, which demonstrated that *SlLCY-b2* was a key node controlling the carotenoid metabolic pathway. Moreover, this speculation was also confirmed by further EMSA experiment that SlBEL11 directly binds to the promoter of *SlLCY-b2* containing **TTGACTTGAC**atagt**GTCA** element (Figure 3). Furthermore, in this work, we showed that SlBEL11 possessed transcriptional repression activity of *SlLCY-b2.*

In addition to the regulation of transcription factors at the molecular level, the accumulation of carotenoids during fruit ripening is also affected by different hormones such as ethylene, auxin, ABA and brassinolide (42, 43). Therefore, it is also possible that SlBEL11 may regulate production of carotenoids including lutein by combined effect of ABA and ethylene. In this study, we also observed the phenomenon of premature senescence of tomato fruits, manifesting in accelerated fruit shedding. This may be due to the increased accumulation of downstream carotenoids providing the raw materials for ABA synthesis, but further experiments are needed to verify the specific mechanism (23, 44).

**FIGURE 6:**
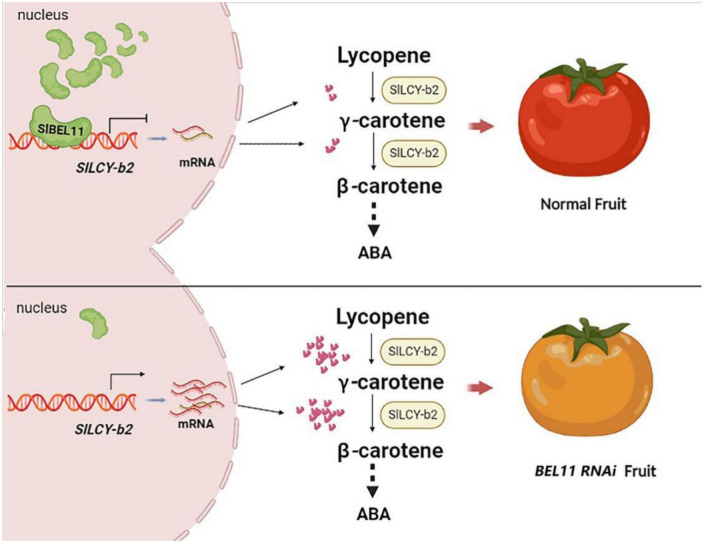
Working principle of the role of SlBEL11 for carotenoid biosynthesis in tomato fruit. SlBEL11 was a transcription factor that acted directly on the promoter of *SlLCY-b2* and suppressed its transcription. When SlBEL11 was silenced, the gene encoding the SlLCY-b2 enzyme that catalyzed lycopene was upregulated, resulting in increased downstream carotenoid accumulation. Ultimately, *SlBEL11 RNAi* tomatoes yielded yellow fruits instead of red ones because of abnormal carotenoid accumulation.

Taken together, our experimental results clarified that the transcription factor SlBEL11 could directly bind to the promoter of the target gene *LCY-b2* and suppress its expression. Total or partial silencing of SlBEL11 will promote the expression of *LCY-b2* gene. In addition, *SlBEL11* gene silencing resulted in changes in the expression of core enzyme genes in the carotenoid metabolic pathway, including *PSY1*, P*SY2*, *PDS*, *ZDS*, *LCY-b1/b2*, *CYC-b*, and *LCY-e*. Among them, the relative gene expression of *LCY-b2* gene was up-regulated most obviously. The upregulation of *LCY-b2* expression contributed to increased carotenoid content in tomato fruit including zeaxanthin, violaxanthin and neoxanthin (Table 1). These evidences suggested one way in which the SlBEL11 transcription factor regulated the accumulation of carotenoids in tomato fruit by directly binding to the *SlLCY-b2* gene to suppress its expression (Figure 6).

## Data availability statement

The original contributions presented in this study are included in the article/Supplementary material, further inquiries can be directed to the corresponding author. Sequence data from this article can be found in GenBank data library under the following numbers: *SlBEL11*, *Solanum lycopersicum* (gene ID: 543692), *Slactin* (gene ID: 101260631), *SlPDS* (gene ID: 544073), *SlPSY1* (gene ID: 543988), *SlPSY2* (gene ID: 543964), *SlZDS* (Gene ID: 543629), *SlCRTISO* (Gene ID: 101267857), *SlLCY-e* (Gene ID: 544129), *SlCYC-b* (Gene ID: 543649), *SlLCY-b1* (gene ID:544104), *SlLCY-b2* (gene ID:101267662), *AtLCY-b*, *Arabidopsis thaliana* (U50739), *OsLCY-b*, *Oryza sativa* (XM_015771748).

## Author contributions

YH: data acquisition and analysis, writing and modification of manuscript, and conduct of experiments. YW: assistance of software installation and use. MZ: instrument appointment. GL and CT: data collection and sorting. XX, YP, XS, and ZZ: methodology. LM: supervision, review of manuscript, acquisition of funds, and guidance for submission. All authors agreed with the above contribution description.
