# Supplementary material for: SlBEL11 affects tomato carotenoid accumulation by regulating SlLCY-b2
Source: Front Nutr. 2022 Dec 22;9:1062006. doi: 10.3389/fnut.2022.1062006 (PMC9814965; doi:10.3389/fnut.2022.1062006)
Supplement: Supplementary file 1 [file Data_Sheet_1.docx]

**
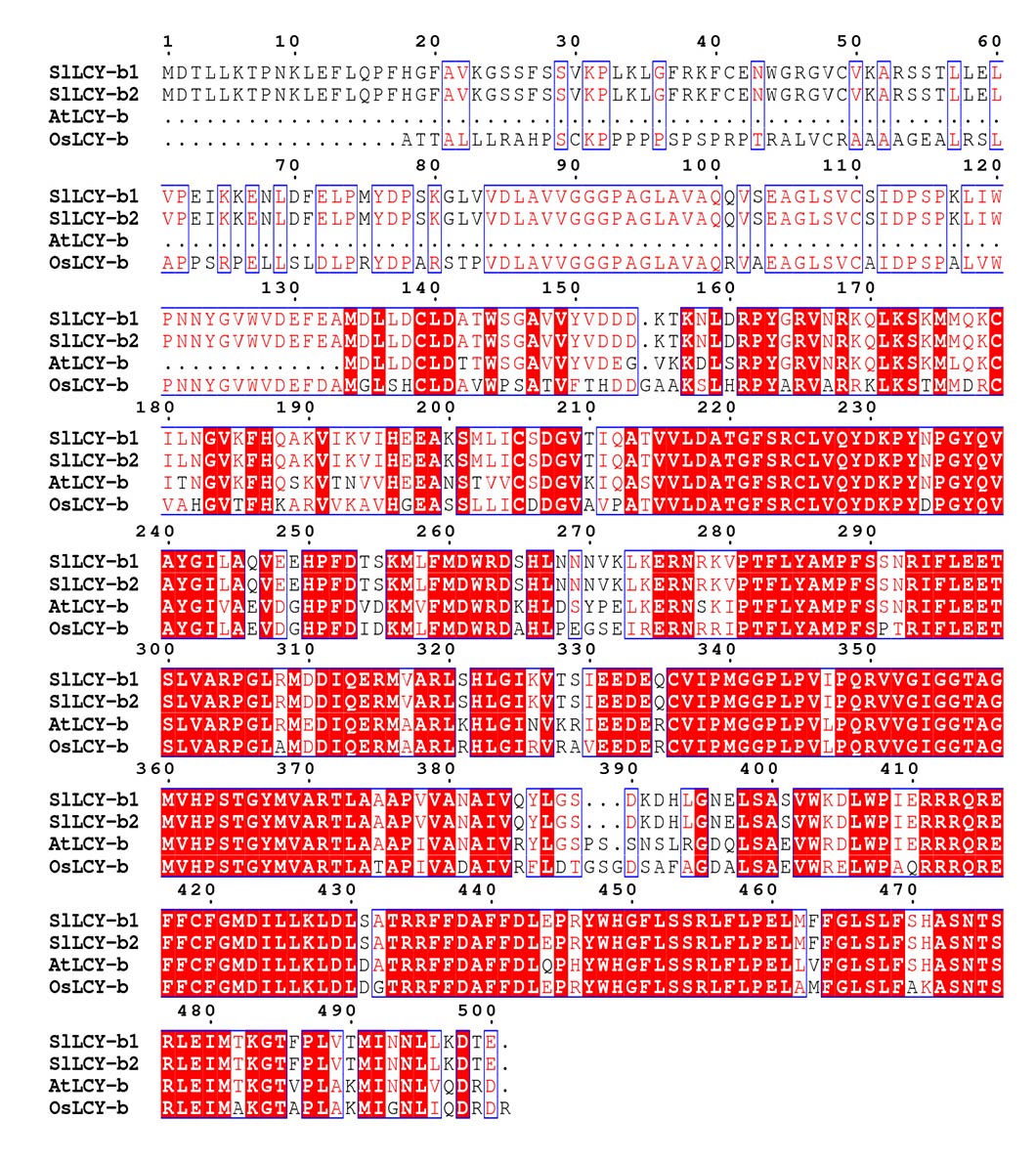
**

**Supplementary Figure 1.** Alignment of the amino acid sequences of *SlLCY-b2* and *LCYbs* genes from other species. The alignments were performed using ClustalX. Red shading and red letters indicate identical and conserved amino acid residues, respectively.

| **Supplementary Table 1**. Primers used in the experiment. | | |
| --- | --- | --- |
| **Assay** | **Name** | **Sequence from 5'to 3'** |
| **VIGS** | ***TRV2-*** ***SlPDS-F*** | **gggacatgcccgggcctcgagTTGAAGCTCAAGATGGGATAAGTG** |
|  | ***TRV2-*** ***SlPDS-R*** | **tggggatccggtaccgagctcGTGAGCTTCTGCTGAAGAGCAA** |
|  | ***TRV2- SlLCY-b1-F*** | **gggacatgcccgggcctcgagTATAATGAATGGTGTTAAATTCCACCA** |
|  | ***TRV2- SlLCY-b1-R*** | **tggggatccggtaccgagctcGTTGGATGAAAATGGCATTGC** |
|  | ***TRV2- SlLCY-b2-F*** | **gggacatgcccgggcctcgagGTTATAAAGGTAATTCATGAGGAAGCTAA** |
|  | ***TRV2- SlLCY-b2-R*** | **tggggatccggtaccgagctcGAGCTACAAGGGAGGTTTCTTCAA** |
|  | ***TRV2- SlLCY-b1+b2-F*** | **taacGTTGGATGAAAATGGCATTGC** |
|  | ***TRV2- SlLCY-b1+b2-R*** | **gccattttcatccaacGTTATAAAGGTAATTCATGAGGAAGCTAA** |
|  | ***Slactin-F*** | **CAGCAGATGTGGATCTCAAA** |
|  | ***Slactin-R*** | **CTGTGGACAATGGAAGGAC** |
| **qRT-PCR** | ***LCY-e –qPCR-F*** | **AACTGATGCTACGATTGAAC** |
|  | ***LCY-e-qPCR-R*** | **ATCTGACGACTGAATAACCT** |
|  | ***SlBEL11-F*** | **GGATTCTAACCGTCCTTCCA** |
|  | ***SlBEL11-R*** | **ATTTGCTTCCATTTCCTTCTCC** |
|  | ***PDS-qPCR-F*** | **CATTGATTATCCAAGACCAGAG** |
|  | ***PDS-qPCR-R*** | **CCAGCAATAACAATCTCCAA** |
|  | ***PSY1-qPCR-F*** | **ATGTCTGTTGCCTTGTTATG** |
|  | ***PSY1-qPCR-R*** | **TTCCACCACCTCTATTGATT** |
|  | ***PSY2-qPCR-F*** | **CAGTAGGATTGATGAGTGTTC** |
|  | ***PSY2-qPCR-R*** | **CTCTGAGTATATTGGTTAGTTGAT** |
|  | ***ZDS-qPCR-F*** | **TCTTGCTGGCTCATATACA** |
|  | ***ZDS-qPCR-R*** | **AGACTCAACTCATCAGATAGG** |
|  | ***LCYb1-qPCR-F*** | **TGAAGCAGGACTCTCTGTTTGT** |
|  | ***LCYb1-qPCR -R*** | **GCCGTATTATCATCAATGTACACT** |
|  | ***LCYb2-qPCR-F*** | **ggaggctgggttatcggtttgc** |
|  | ***LCYb2-qPCR-R*** | **gttttatcatcatcgacataaacaa** |
|  | ***CYCb-qPCR-F*** | **TGTTATTGAGGAAGAGAAATGTGTGAT** |
|  | ***CYCb-qPCR-R*** | **TCCCACCAATAGCCATAACATTTT** |
|  | ***CRTISO-qPCR-F*** | **TTTTGGCGGAATCAACTACC** |
|  | ***CRTISO-qPCR-R*** | **GAAAGCTTCACTCCCACAGC** |
